# Cost of illness and economic burden of chronic lymphocytic leukemia

**DOI:** 10.1186/1750-1172-8-32

**Published:** 2013-02-20

**Authors:** Carl Rudolf Blankart, Taika Koch, Roland Linder, Frank Verheyen, Jonas Schreyögg, Tom Stargardt

**Affiliations:** 1 Hamburg Center for Health Economics, Universität Hamburg, Esplanade 36, D-20354, Hamburg, Germany; 2 WINEG—Scientific Institute of the Techniker Krankenkasse (TK) for Benefit and Efficiency in Health Care, Bramfelder Straße 140, D-22305, Hamburg, Germany

**Keywords:** CLL, Routine data, Administrative data, Economic evaluation, Case–control design, Elixhauser comorbidity groups

## Abstract

**Background:**

Chronic lymphocytic leukemia (CLL) is a slowly progressing but fatal disease that imposes a high economic burden on sickness funds and society. The objective of this study was to analyze and compare the direct and indirect costs of CLL in Germany from the perspective of the sickness funds and society and analyze the burden of the disease.

**Methods:**

Using a database of 7.6 million enrolled individuals, we identified 4198 CLL patients in 2007 and 2008. The costs attributable to CLL were estimated using a case–control design with a randomly selected control group of 150 individuals per combination of age and sex. We used the bootstrap approach to estimate uncertainties in costs estimated. We employed generalized estimating equation regression models and count data models to test for differences in costs and healthcare utilization.

**Results:**

The cost attributable to CLL for each prevalent case amounts to €4946 from the payer’s perspective and €7910 from a societal perspective. Inpatient hospital stays and pharmaceuticals are the main cost drivers of the disease. The economic burden of disease in Germany was estimated to be approximately €201 million per year for the sickness funds and €322 million for society.

**Conclusions:**

Compared with common diseases, such as diabetes or COPD, the economic burden of CLL is considerably lower. However, the cost of treatment per case is about twice as high as the cost per case for these common diseases, even though treatment is only performed in the later stages of CLL. With new healthcare technologies, the aging population, and the increasing incidence of the disease, it is likely that the economic burden of the disease will continue to grow.

## Background

B-cell chronic lymphocytic leukemia (CLL) is a rare form of cancer that most often appears in older patients. The median age at first diagnosis is 64 years, with an expected survival of more than 10 years [1]. The prevalence is estimated to be 3 per 10,000 people [2], with an incidence of 0.46 per 10,000 people per year [3]. Although CLL is the most common type of adult leukemia in the western hemisphere [4,5], it is classified as a rare disease according to the standards of the EU (where the prevalence criterion for a rare disease is set at <5 per 10,000 people) and U.S. (<7.5 per 10,000 people) [6].

The vast majority of the literature on CLL compares different medical treatment options or evaluates comorbidities and their influence on the progression of the disease, but there are few comprehensive cost studies [5,7]. The literature written from an economic perspective primarily consists of cost-effectiveness studies that focus only on the cost differences between pharmacological treatments. This study provides robust and reliable information on the burden of a rare disease from the sickness fund perspective and societal perspective, using a large sample of CLL patients. First, we calculated costs attributable to CLL, defined as difference between costs of a CLL patient and costs of an average enrolled individual with the same age and sex. Additionally, cost differences in various cost categories and indicators for health services utilization were analyzed using generalized estimating equation regression (GEE) [8] and count data models. We used the bootstrap approach [9] for uncertainty estimation. In this study, we demonstrated a sophisticated economic approach to analyzing the burden of chronic disease using administrative data.

## Methods

### Study sample and study design

We obtained access to data from the Techniker Krankenkasse, a large German sickness fund that covers 7.6 million people (i.e., approximately 9% of Germany’s population in 2008). We collected panel data for two different groups of patients in 2007 and 2008. First, we selected all enrolled persons who had been given a reliable ICD-10 diagnosis of C91.1 (the CLL group). Second, we randomly selected 150 male and female enrolled individuals for every year of age between 0 and 100, i.e., 150 × 2 × 100 individuals. To form the control group, we selected 150 individuals with the same age and sex per CLL patient, i.e., we created a control group consisting of 4198 × 150 observations while we used the age- and sex-specific controls multiple times if one age and sex group contained more than one prevalent subject. By comparing the differences between the two groups, we were able to calculate the costs attributable to CLL. To be included in our study, all patients were required to be continuously enrolled in the calendar year of 2007 or 2008 or in both years, with the exception of patients who died during the observation period.

The dataset included socio-demographic information (i.e., age, sex, employment status, entitlement to a regular or occupational disability pension, etc.) and the direct medical and non-medical costs to the payer. We also obtained information on the participants’ inpatient and outpatient diagnoses, sick-leave days, hospital days, physician contacts, inpatient stays, and prescriptions filled.

### Measurement of costs from the perspective of the sickness funds

The direct medical costs included the costs of in- and outpatient care, pharmaceuticals, nursing care at home, medical aids, services from non-physicians, and rehabilitation. In the absence of specific data on dental costs, we included the average dental cost across all sickness funds in Germany for all patients [10]. The direct non-medical costs reimbursed by sickness funds comprised the expenses paid for sick leave, travel expenses for physician visits, and the cost of transporting patients via ambulance. Administration costs were also included using the average administrative spending amount across all sickness funds [11] to increase the generalizability of results.

### Measurement of costs from the societal perspective

To calculate the costs from the societal perspective, we made several adjustments to the data. First, we calculated the indirect costs (i.e., productivity losses) following the human capital approach [12]. We calculated the productivity losses at the patient level by multiplying the gross income (adjusted by sex and year [13]) by the number of sick-leave days. To consider disease-related unemployment, we corrected our data to reflect the average proportion of working household members reported by the federal statistical office.

Second, to better reflect the costs, we considered the co-payments made by the patients for direct and indirect medical costs. In our analysis, we included the following co-payments at the patient level for all participants over 18 years of age: (i) €10 per outpatient consultation with a physician per quarter, up to a maximum of €40 per year; (ii) €5 per prescription priced below €50, 10% per prescription priced above €50 and below €100, and €10 per prescription priced above €100; and (iii) €10 per day in the hospital. We also took into account that the total annual co-payment is limited to a maximum of 2% of the patient’s gross income or in the case of a chronic disease such as CLL, 1% of gross income. To adjust co-payments to reflect these limits, we used average wages adjusted by sex and year [13]. The corrections were applied proportionally to each category of medical costs. Third, the pharmaceutical costs were corrected to reflect the value-added tax (19%), and fourth, the hospital costs were multiplied by 1.047 to reflect the hospitals’ investment costs not covered by DRGs [14].

### Statistical analysis

First, we calculated annual means for each cost category and the non-cost indicators for both the CLL and control groups. We used the bootstrap approach [9] to estimate uncertainties in costs estimated, i.e., we created 1000 datasets by drawing randomly with replacement from the original dataset. The 2.5 and the 97.5% quantiles of the bootstrap distribution were used as the limits of the 95% confidence interval. We then calculated the total costs attributable to CLL from sickness fund as well as from societal perspective. Second, as most of our data were not normally distributed, we analyzed the differences in means by using GEE regression models. We employed a correlation matrix with an autoregressive structure in all our models to control for correlations due to repeated measurements. We assumed a gamma distribution and used log-link function for the analysis of all cost categories. Zero-truncated gamma models were estimated for all cost categories where the dependent variable was zero for at least one observation as the dependent variable must not be zero in case of gamma-specified regression models. Thus, we split the analysis into two parts, i.e., first we analyze if there is a difference between zero and non-zero cost using a binary variable and second we analyze the conditional data using a gamma specified GEE model. The differences between the indicators for health care utilization, such as the days in the hospital or the number of prescriptions, were tested using count data models. Based on the results of the Vuong test [15], we considered Poisson or negative binominal regression models for modeling the number of physician contacts, the number of inpatient stays, and the number of prescriptions filled. Sick-leave days were modeled with a zero-inflated negative binomial regression model (ZINB) because of excess zeros.

Regarding explanatory variables, we included age and age-squared, as well as dummy variables for CLL vs. control, sex, and the 31 Elixhauser comorbidity groups [16,17], in all our models. We allowed the Elixhauser comorbidity groups to interact with age and age-squared because the costs associated with comorbidities might increase or decrease with age. For example, patients suffering from diabetes are treated with relatively inexpensive drugs during the early stages of the disease in their younger years; at a more advanced stage of the disease, they may develop complications and need expensive surgery; and finally, at an older age, diabetes treatment may be reduced because of other deadly comorbidities. In addition, we included the interaction of age and age-squared with sex. The data were Winsorized at the upper 0.01% level to account for outliers.

Next, we performed an analysis of the pharmaceuticals administered. In addition to chemotherapeutic drugs, we analyzed anti-infective drugs because CLL patients have an increased risk of contracting bacterial infections due to the leukemia itself (humoral and cellular immune dysfunction) and immunosuppression treatment [18]. We differentiated between the drug classes using the Anatomical Therapeutic Chemical Classification System (ATC), and we evaluated the costs for the following classes: (i) pharmaceuticals acting on blood and blood-forming organs (ATC B); (ii) anti-infectives for systemic use (ATC J); (iii) antineoplastic and immunomodulating agents (ATC L); (iv) cytostatic drugs individually prepared in the pharmacy; and (v) other pharmaceuticals. To conduct a sensitivity analysis, we performed all calculations for the years 2007 and 2008 separately as subgroup analyses.

Because CLL is more common in older individuals but very young patients are also affected, we expected the costs of the disease to differ by age group. For example, healthcare providers may put more effort into treating younger patients than older patients. However, the higher costs for these treatments may be counteracted by the fact that many young CLL patients are in the ‘Binet A’ stage (watch and wait), which does not require expensive treatments, such as chemotherapy or hospitalization. To explore this issue, we calculated the average costs for different age classes; we grouped all observed individuals into 5-year age classes (0–5, 6–10, …, 96–100) and calculated the average total costs by age class. We also plotted the mean cost for each age group and also created a scatter plot of the frequencies of observations.

### Calculation of the economic burden of the disease

We calculated the economic burden of the disease for Germany by extrapolating our results for the 7.6 million individuals enrolled at the Techniker Krankenkasse to the entire German population (81.8 million). To control for uncertainty, we also calculated the economic burden using the upper and lower boundaries of the confidence intervals of the total costs. To account for potential differences in the prevalence of the disease between our population and the whole of Germany, we also calculated the burden of the disease based on the prevalence figures we obtained from the literature (i.e., data from a study by Reis et al. [19], the Orphanet database [2], and the SEER database of the US National Cancer Institute [3]).

## Results

We identified a total of 4198 patients with CLL in 2007 and 2008. Of these patients, we were able to follow 3321 patients for both years, while 877 patients could be observed for only one calendar year. This resulted in 7519 observations for our analysis, and based on these figures, we calculated an average prevalence of 4.9 per 10,000 people for 2007/2008. The baseline characteristics are shown in Table 1. The CLL group and control group had an average age of 66.1 years (standard deviation 13.2), and the majority of the patients were male (66.9%). The youngest CLL patient in the sample was a one-year-old boy, while the oldest was a 99-year-old woman. For the CLL group, the mortality rate was 4.88% per year, while the mortality rate was 1.72% in the control group. Not including common diseases of the elderly (e.g., hypertension) or non-cancer-related comorbidities (e.g., drug abuse), the patients in the CLL group had significantly more comorbidities across all of the Elixhauser comorbidity groups. Similarly, the Elixhauser score was 10.0 for the CLL group and therefore significantly higher (p < 0.001) than the score of 5.2 for the control group.

**Table 1:** Baseline characteristics and comorbidities

	**CLL group**		**Control group**		
	**Mean**	**STD**	**Mean**	**STD**	**p-Value**
**Baseline characteristics**					
Age	66.1	(13.2)	66.1	(13.2)	
Sex (male = 1)	66.9%		66.9%		
Mortality (per calendar year)	4.9%		1.7%		
**Elixhauser comorbidity groups**					
Congestive heart failure	12.7%		10.3%		<.001
Cardiac arrhythmias	20.9%		18.4%		<.001
Valvular disease	10.3%		8.5%		<.001
Pulmonary circulation disorders	3.0%		2.0%		<.001
Peripheral vascular disorders	13.2%		11.8%		<.001
Hypertension uncomplicated	52.6%		53.3%		0.214
Hypertension complicated	9.1%		8.4%		0.032
Paralysis	2.4%		2.1%		0.030
Other neurological disorders	4.5%		4.4%		0.469
Chronic pulmonary disease	23.8%		18.4%		<.001
Diabetes uncomplicated	19.5%		18.0%		0.001
Diabetes complicated	8.0%		7.1%		0.003
Hypothyroidism	9.2%		7.6%		<.001
Renal failure	10.4%		7.9%		<.001
Liver disease	14.1%		12.2%		<.001
Peptic ulcer disease excluding bleeding	2.4%		2.0%		0.010
AIDS/HIV	0.2%		0.1%		<.001
Lymphoma	27.7%		0.8%		<.001
Metastatic cancer	9.6%		3.2%		<.001
Solid tumor without metastasis	22.9%		17.1%		<.001
Rheumatoid arthritis/collagen vascular diseases	8.1%		6.6%		<.001
Coagulopathy	11.2%		3.4%		<.001
Obesity	9.6%		10.2%		0.062
Weight loss	3.2%		1.1%		<.001
Fluid and electrolyte disorders	9.3%		4.5%		<.001
Blood loss anemia	0.5%		0.4%		0.030
Deficiency anemias	4.5%		2.9%		<.001
Alcohol abuse	1.7%		2.2%		0.003
Drug abuse	0.5%		0.4%		0.136
Psychoses	1.0%		0.8%		0.018
Depression	17.3%		13.4%		<.001
**Elixhauser score**	10.0	(10.5)	5.2	(7.9)	<.001

*CLL* Chronic lymphocytic leukemia, *STD* Standard deviation.

Healthcare utilization differed significantly between the CLL group and control group (Table 2). We found that on average, the CLL patients were absent from work for 7.6 days, while the individuals in the control group spent 3.1 days on sick leave. The CLL patients contacted a physician significantly more often (p < 0.001), stayed in the hospital more often (p < 0.001), and received drug prescriptions more often (p < 0.001) than their age- and sex-adjusted counterparts.

**Table 2:** Indicators for healthcare utilization

	**CLL group**		**Control group**		**Attributable to CLL**			
	**Mean**	**STD**	**Mean**	**STD**	**Lower limit (2.5%)**	**Mean**	**Upper limit (97.5%)**	**p-Value**
Sick leave days (paid and unpaid days)	7.6	(35.5)	3.1	(19.6)	3.6	4.4	5.2	<.001 (Logit) <.001 (NB)^a^
Physician contacts	14.7	(7.5)	9.4	(6.6)	5.1	5.3	5.4	<.001^b^
Inpatient stays	0.95	(2.10)	0.36	(0.96)	0.54	0.59	0.63	0.007^b^
Average length of stay (if hospitalized)	9.4		8.8			0.5		
Prescriptions	18.2	(19.3)	12.8	(14.4)	5.0	5.4	5.9	<.001^a^
Average prescription cost	148.2		44.5			103.8		

*CLL* chronic lymphocytic leukemia*, STD* Standard deviation.

a) Zero-inflated negative binomial specified model; b) Negative binominal specified model.

The costs for various categories of expenses from the sickness fund perspective and societal perspective are presented in Table 3. The total average cost for a CLL patient was €9753 per year, compared with €4807 for a control group participant of the same age and sex. The total average costs between the CLL patients and the control group differed significantly (p < 0.001). The additional cost of CLL thus amounted to €4946 for each patient. After performing the bootstrap, we identified a 95%-percentile interval of €4612 to €5499 for total costs attributable to CLL from sickness fund perspective. The costs of inpatient care and pharmaceuticals were the main drivers of direct medical costs for CLL patients, amounting to €3453 and €2699, respectively. The pharmaceutical costs for CLL patients were approximately five-fold higher than those for the control group. However, the proportion of CLL patients that get pharmaceuticals prescribed does not differ from the control group (p = 0.816), but the pharmaceutical costs per year were significantly higher (p < 0.001) if pharmaceuticals were prescribed. Chemotherapy drugs (i.e., antineoplastic and immunomodulating agents) (€835), cytostatic drugs individually prepared in the pharmacy (€504), and anti-infective drugs for systemic use (€517) represented the largest fractions of pharmaceutical costs (see Table 4). Inpatient costs of CLL patients and control group did not significantly differ (p = 0.267) if they were hospitalized. However, CLL patients had a higher risk to have at least one inpatient stay per year (p < 0.001). The proportion of CLL patients that did contact an outpatient physician did not differ from the control group (p = 0.873). However, those patients that do visit an outpatient physician were significantly more costly (p < 0.001). The economic burden of the disease for the sickness funds in Germany was estimated to be €201 million per year with a confidence interval between €187.6 and €223.6 million. Depending on the prevalence assumptions used for the calculations, the economic burden from the point of view of the sickness funds varied between €122.0 and €201.1 million per year (see Table 5).

**Table 3:** Average costs per person per year from the sickness fund perspective and societal perspective

	**Sickness fund perspective**								**Societal perspective**							
	**CLL group**		**Control group**		**Costs attributable to CLL**				**CLL group**		**Control group**		**Costs attributable to CLL**			
	**Mean**	**STD**	**Mean**	**STD**	**Lower limit (2.5%)**	**Mean**	**Upper limit (97.5%)**	**p-Value**	**Mean**	**STD**	**Mean**	**STD**	**Lower limit (2.5%)**	**Mean**	**Upper limit (97.5%)**	**p-Value**
**Direct medical costs**	**€9331**	**(19,795)**	**€4533**	**(15,085)**	**€4365**	**€4798**	**€5233**	**<.001**^**a**^	**€9221**	**(19,569)**	**€4617**	**(15,080)**	**€4173**	**€4604**	**€5038**	**<.001**^**a**^
Outpatient cost	€1699	(3748)	€1246	(4304)	€370	€453	€544	0.873 (Logit)<.001 (Gamma)^b^	€1735	(3747)	€1281	(4305)	€372	€455	€545	0.545 (Logit)
																<.001 (Gamma)^b^
Inpatient cost	€3453	(11,793)	€1109	(4178)	€2069	€2344	€2602	<.001 (Logit)0.267 (Gamma)^b^	€3663	(12,399)	€1185	(4425)	€2190	€2478	€2750	<.001 (Logit)
																0.156 (Gamma)^b^
Pharmaceuticals	€2699	(7469)	€568	(1989)	€1963	€2131	€2297	0.816 (Logit)<.001 (Gamma)^b^	€2342	(6308)	€541	(1699)	€1659	€1801	€1941	0.843 (Logit)
																<.001 (Gamma)^b^
Nursing care (at home)	€858	(9951)	€1035	(11,822)	-€382	-€177	€52	0.104 (Logit)0.900 (Gamma)^b^	€858	(9951)	€1035	(11,822)	-€382	-€177	€52	0.104 (Logit)
																0.900 (Gamma)^b^
Dentistry	€156	(0)	€156	(0)		€0			€156	(0)	€156	(0)		€0		
Other	€466	(2111)	€418	(2082)	€4	€48	€98	0.508 (Logit)0.813 (Gamma)^b^	€466	(2111)	€418	(2082)	€4	€48	€98	0.508 (Logit)
																0.813 (Gamma)^b^
**Direct non-medical costs**	**€231**	**(1558)**	**€84**	**(754)**	**€113**	**€147**	**€191**	**<.001 (Logit)** **0.116 (Gamma)**^**b**^	**€117**	**(759)**	**€58**	**(476)**	**€44**	**€59**	**€78**	**<.001 (Logit)**
																**0.923 (Gamma)**^**b**^
Sick pay	€114	(1340)	€26	(576)	€60	€88	€121	0.039 (Logit)0.072 (Gamma)^b^	€0		€0			€0		
Other	€117	(759)	€58	(476)	€44	€59	€78	<.001 (Logit)0.923 (Gamma)^b^	€117	(759)	€58	(476)	€44	€59	€78	<.001 (Logit)
																0.923 (Gamma)^b^
**Administration**	**€191**	**(0)**	**€191**	**(0)**		**€0**			**€191**	**(0)**	**€191**	**(0)**		**€0**		
**Indirect cost**	**€0**		**€0**			**€0**			**€4410**	**(8233)**	**€1164**	**(4546)**	**€3073**	**€3246**	**€3400**	**0.009 (Logit)**
																**<.001 (Gamma)**^**b**^
**Total cost**	**€9753**	**(20,199)**	**€4807**	**(15,219)**	**€4612**	**€4946**	**€5499**	**<.001**^**a**^	**€13,939**	**(22,516)**	**€6030**	**(16,037)**	**€7434**	**€7910**	**€8392**	**<.001a**

*CLL* Chronic lymphocytic leukemia, *STD* Standard deviation.

a) Gamma specified model; b) Zero-truncated gamma model.

**Table 4:** Pharmaceutical costs per year by therapeutic class

	**CLL group**		**Control group**	
**Costs per year**	**Mean**	**STD**	**Mean**	**STD**
ATC B - Blood and blood forming organs	€143	(1123)	€42	(545)
ATC J - Anti-infectives for systemic use	€517	(2955)	€21	(396)
ATC L - Antineoplastic and immunomodulating agents	€835	(4906)	€77	(1063)
Cytostatic drugs	€504	(2392)	€41	(835)
Other	€700	(2348)	€386	(996)
**Total pharmaceuticals costs**	**€2699**	**(7469)**	**€568**	**(1980)**

**Table 5:** The burden of disease in Germany based on prevalence data from the literature

				**Sickness fund perspective**			**Societal perspective**				
	** Year**	**Study origin**	**Prevalence**	**Lower limit (2.5%)**	**Burden (mean)**	**Upper limit (97.5%)**	**Direct costs**	**Indirect costs**	**Lower limit (2.5%)**	**Burden (mean)**	**Upper limit (97.5%)**
This study	2007/2008	Germany	4.95	€187.6 m	**€201.1 m**	€223.6 m	€189.7 m	€132.0 m	€302.3 m	**€321.7 m**	€341.3 m
Orphanet database^2^	2011	not stated/global	3.00	€113.8 m	**€122.0 m**	€135.6 m	€115.0 m	€80.1 m	€183.4 m	**€195.1 m**	€207.0 m
Reis et al.^1^	2000	Germany	4.53				€262.0 m	€52.0 m		**€314.0 m**	
Reis et al. (adj.)^2^	2007/2008	Germany	4.53	€171.8 m	**€184.3 m**	€204.9 m	€173.7 m	€121.0 m	€277.0 m	**€294.7 m**	€312.7 m
SEER database^2^	2008	USA	3.47	€131.6 m	**€141.1 m**	€156.9 m	€133.1 m	€92.6 m	€212.1 m	**€225.7 m**	€239.5 m

1) as reported by Reis et al. (2006); 2) calculated based cost data of this study.

**Figure 1:**
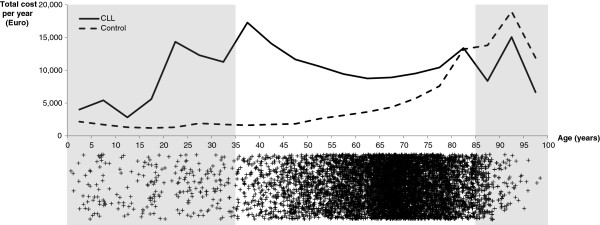
Total annual costs for the CLL group and control group by age.

The average costs for various cost categories per year by age from the sickness fund perspective are shown in Figure 1. The scatter plot below the cost plot represents the frequency of observations. As the frequency was low for patients younger than 35 or older than 85 years of age, we considered the costs per age class to be reliable only for the age groups between 35 and 85. The average yearly cost for each CLL patient from the sickness fund perspective decreased with increasing age until the age of 60–65 and thereafter increased again. The additional cost burden of caring for CLL patients compared with the control group peaked between the ages of 35 and 40.

From the societal perspective, the total annual cost for a CLL patient amounted to an average of €13,939 compared with €6030 for an individual in the control group. The additional cost of CLL thus amounted to €7910 per patient. After performing the bootstrap, we identified a 95%-percentile interval of €7434 to €8392 for total costs attributable to CLL from societal perspective. The main cost drivers were indirect costs, amounting to €4410 for CLL patients and €1164 for the control group. The economic burden of the disease amounted to €321.7 million for Germany, with €189.7 million in direct costs and €132.0 million in indirect costs. The confidence interval was estimated to be between €302.3 and €341.3 million. Depending on the prevalence assumptions used for the calculations, the economic burden from the societal perspective varied between €195.1 and €321.7 million per year (Table 5).

## Discussion

In this study, we presented detailed cost data for CLL in Germany and data on the economic burden of the disease. We compared the costs of a large sample of CLL patients with a randomly drawn sample, consisting of 150 individuals per CLL patient, selected by age and sex. The detailed cost data from the payer perspective and societal perspective complement existing studies on the treatment of CLL and cost-effectiveness analyses of different chemotherapy drugs.

Cost of illness was analyzed using a matched control, prevalence-based approach. The matched control approach achieves that disease specific costs are isolated by subtracting out the costs of matched patients and it provides more accurate estimates compared to methods that sum all medical or diagnosis specific costs [20]. Application of a regression method was not considered to be feasible as our analysis is primarily based on administrative data. Administrative data does not allow controlling for unobservable differences, such as genetic factors that contribute to the etiology of CLL [21], and therefore regression analysis would lead to biased results [20]. The prevalence-based approach was considered appropriate to represent the total current economic burden of CLL [22,23].

Compared with earlier findings [2,3], we identified a considerably higher prevalence of 4.9 per 10,000 individuals. Considering that the EU threshold for a rare disease is set at 5 per 10,000 individuals, CLL is, according to our sample, just below the threshold of a rare disease, but it is much more prevalent than the 3.47 per 10,000 reported by the US National Cancer Institute [3] or the 3.0 per 10,000 reported by Orphanet [2]. However, the data from Reis et al. suggest a similar prevalence of 4.5 per 10,000 people (861 CLL patients in a database of 1.9 million individuals) in Germany. The estimated prevalence has a substantial impact on the estimation of the economic burden of the disease. Taking into account that CLL progresses slowly and is diagnosed late, the prevalence figures we have presented may be underestimated because of the potentially high number of non-diagnosed cases.

From the sickness fund perspective, the costs attributable to CLL for a single patient were €4946, using an average patient of the same age and sex for comparison. Much of this cost difference can be explained by higher inpatient and pharmaceutical costs. According to our data, an average CLL patient had 0.95 hospital stays per year, with an average stay of 9.4 days, while we found 0.36 inpatient stays with an average length of 8.8 days in the control group. Therefore, an average CLL patient stayed in the hospital 8.9 days per year, compared with 3.0 days in the control group, for a total of 5.9 hospital days per patient per year attributable to CLL. Interestingly, Reis et al. reported that an average lymphoma patient spends 27 days (attributable to their disease) in the hospital per year, with an average cost per day of €139 [19]. In addition, they reported attributable inpatient costs of approximately €2000 for an average CLL patient, which is equivalent to 14.4 hospital days, if hospital days are re-calculated based on the average hospital costs per day for lymphoma patients reported in their study. While the inpatient costs are comparable to our finding of €2344, the length of inpatient stays is much higher. Therefore, hospital costs per day seem to have increased substantially since 2000, while the average length of inpatient stay has decreased. This change may have been induced by the change in the hospital reimbursement system from per diem amounts to reimbursement for each case (i.e., DRGs) and improvements in healthcare technology [24]. New healthcare technologies may have increased costs but reduced the length of stay.

Regarding the economic burden of CLL on society, our results are comparable to the findings of Reis et al. (€321.7 million (our study) vs. €314 million (Reis et al.)). However, we calculated lower direct costs (€189.7 million vs. €262.0 million) and higher indirect costs (€132.0 million vs. €52 million). Regarding these different results, it should be mentioned that the costs calculated by Reis et al. are based on the year 2000 and are not discounted. Second, as mentioned above, there have been major changes in the reimbursement system, especially in the inpatient sector, that most likely led to a reduction in the length of stay. This development may have compensated for the increases in inpatient costs due to new health technologies. Third, advances in healthcare technology in the area of pharmaceutical care, such as chemotherapeutic drugs, have increased costs. Compared with the results from Reis et al., we found pharmaceutical costs to be more than twice as high. Similarly, sales for rituximab, a chemotherapeutic drug for the treatment of CLL, have more than tripled since 2000 [25,26].

The costs for an average CLL patient decrease until the age of 65 and increase again with advancing age, while the costs for the control group increase steadily until the age of 85. Accordingly, the difference in the cost of CLL compared with the control group decreases to zero at 85. One possible reason is that costly procedures, such as stem cell transplantation, are used only for patients under the age of 66. In addition, sick pay is only paid for employed patients, who are mainly individuals under the age of 65. With increasing age, the cost curve of the control group asymptotically converges with that of CLL patients due to age-related comorbidities. Bearing in mind that CLL is a slow-progressing disease, we conclude that younger patients are more extensively treated for CLL to increase their chances of survival, while the treatment effort attributable to CLL is reduced for older patients because CLL might not decrease survival for these patients.

In comparison with other chronic diseases, the overall economic burden of CLL is low. Although we have to consider that a comparison of cost of illness studies is subject to limitations, especially if different methods are used [20,22], a discussion with the cost of common diseases is helpful in interpreting the results. Widespread and common chronic diseases, such as diabetes (€22,288 million in 2001 [27]) and cardiovascular disease (€11,048 million in 2003 [28]), are among the most expensive diseases in Germany. As CLL is a rare disease, its burden is approximately 1.4% of the burden of diabetes. However, the prevalence of CLL is 0.71% of the prevalence of diabetes, indicating twice the level of spending per prevalent CLL case. Nevertheless, the costs per prevalent CLL case are lower than those for cancers of other sites, such as lung cancer (US$19,196 per prevalent case, approx. €18,011), prostate cancer (US$8250, approx. €7741), or ‘other cancer sites’ (US$12,131, approx. €11,382), reported by Yu et al. for the year 1999 [29]. Because cancers of these sites progress more rapidly than CLL, this finding is unsurprising. However, while the treatment costs in Binet ‘stage 1’ (watch and wait) might be considerably lower, the costs in later stages might exceed those of lung or prostate cancer.

### Limitations

This study is based on administrative data. Administrative data have the advantage of providing a holistic overview of an entire population, covering all outpatient and inpatient care [30]. It is, however, data collected only for reimbursement purposes. Therefore, administrative data lack certain important clinical data (e.g., tumor stage). Considering the typical progression of the disease, it is likely that the costs for CLL differ substantially by stage. Therefore, further research may focus on evaluating CLL costs at various stages of the disease.

When compared with the average sick pay reimbursed by a CLL patient’s sickness fund per year (€114), the productivity losses calculated with the human capital approach (€4410) may still be underestimated because the sickness fund pays only after six weeks of sick leave, while the first six weeks are paid by the employer. Thus, not every claim for being unable to work will be recorded in the sickness funds’ databases. Although this affects both groups, it causes a greater downward bias for the total indirect costs of CLL than the control group.

Another limitation arises from the use of annual cost as response variable in the regression models. As mortality of CLL patients is substantially higher compared to the control group, costs analyzed are biased downward by insured living less than 365 days. However, as healthcare costs are also increased by proximity to death [31], the overall effect of death, which we cannot control for, is lessened.

Assessing uncertainty in cost of illness studies is of particular importance. Within this study we use the bootstrap approach to account for uncertainty. Other approaches, such as the Cholesky decomposition method or the error propagation law, deliver similar results [32]. However, uncertainty assessed only refers to stochastic uncertainty. Although we assessed the impact of different prevalence assumptions on the burden of disease, we cannot account for other causes of uncertainty, such as systematic upcoding [33].

## Conclusions

Our results showed that CLL imposes a high economic burden primarily driven by inpatient and pharmaceutical costs. From the societal perspective, the highest costs arise from productivity losses. The substantial increase in pharmaceutical costs compared with previous studies and the shift to shorter hospital stays [19] are attributable to the rapid emergence of new healthcare technologies in the treatment of CLL. With higher survival rates because of new healthcare technologies, as well as an aging population and the increasing incidence indicated by SEER data [3], it is likely that the economic burden of the disease will continue to increase in the future.

## Competing interests

Roland Linder and Frank Verheyen are employed by the Techniker Krankenkasse, a large German sickness fund, and may have potential competing interests according to the guidelines of the International Committee of Medical Journal Editors.

Carl Rudolf Blankart, Taika Koch, Jonas Schreyögg, and Tom Stargardt declare that they have no competing interests.

## Authors’ contributions

CRB and TK collected and refined the data. CRB, TK, JS and TS participated in the design of the study and performed the statistical analysis. CRB, RL, FV, JS, and TS conceived of the study, and participated in its design and coordination and helped to draft the manuscript. All authors read and approved the final manuscript.
